# Effects of Dopamine D2-Like Receptor Antagonists on Light Responses of Ganglion Cells in Wild-Type and P23H Rat Retinas

**DOI:** 10.1371/journal.pone.0146154

**Published:** 2015-12-30

**Authors:** Ralph Jensen

**Affiliations:** VA Boston Healthcare System, Mail Stop 151E, 150 South Huntington Avenue, Boston, Massachusetts 02130, United States of America; Oregon Health & Science University, UNITED STATES

## Abstract

In animal models of retinitis pigmentosa the dopaminergic system in the retina appears to be dysfunctional, which may contribute to the debilitated sight experienced by retinitis pigmentosa patients. Since dopamine D2-like receptors are known to modulate the activity of dopaminergic neurons, I examined the effects of dopamine D2-like receptor antagonists on the light responses of retinal ganglion cells (RGCs) in the P23H rat model of retinitis pigmentosa. Extracellular electrical recordings were made from RGCs in isolated transgenic P23H rat retinas and wild-type Sprague-Dawley rat retinas. Intensity-response curves to flashes of light were evaluated prior to and during bath application of a dopamine D2-like receptor antagonist. The dopamine D2/D3 receptor antagonists sulpiride and eticlopride and the D4 receptor antagonist L-745,870 increased light sensitivity of P23H rat RGCs but decreased light sensitivity in Sprague-Dawley rat RGCs. In addition, L-745,870, but not sulpiride or eticlopride, reduced the maximum peak responses of Sprague-Dawley rat RGCs. I describe for the first time ON-center RGCs in P23H rats that exhibit an abnormally long-latency (>200 ms) response to the onset of a small spot of light. Both sulpiride and eticlopride, but not L-745,870, reduced this ON response and brought out a short-latency OFF response, suggesting that these cells are in actuality OFF-center cells. Overall, the results show that the altered dopaminergic system in degenerate retinas contributes to the deteriorated light responses of RGCs.

## Introduction

Retinitis pigmentosa is an inherited retinal disorder caused by various genetic mutations, many of which encode proteins exclusively expressed in rod photoreceptors [1]. These mutations cause the death of rod photoreceptors and secondary death of cones. With the loss of photoreceptors, substantial remodeling occurs early in the disease process in bipolar cells and horizontal cells which are postsynaptic to photoreceptors [2–6]. Structural and neurochemical changes have also been reported in amacrine cells [7–10], which are postsynaptic to bipolar cells.

A subclass of amacrine cells uses the neurotransmitter dopamine. These dopaminergic amacrine cells are believed to play an important role in neural processes related to changes in ambient light levels [11]. In degenerate retinas, dopaminergic amacrine cells appear to be dysfunctional. In the dystrophic RCS rat model of retinitis pigmentosa, rod-driven horizontal cells are unresponsive to the D1 receptor antagonist SCH 23390 [12]. Since D1 receptors were shown to still be functional in these cells, the authors suggest that there is a significant reduction in the amount of dopamine released in retinas of these rats compared to control animals. In the rds mouse model of retinitis pigmentosa, dopamine synthesis and utilization are reduced [13], suggesting again a low release of dopamine from dopaminergic neurons. In the rd1 mouse model of retinitis pigmentosa, Atkinson et al. [14] found from patch-clamp recordings of retinal dopaminergic neurons that these cells exhibit less spontaneous bursting, which as they suggested could result in a decreased release of dopamine. Interestingly, they also reported in the same study that light-evoked responses from dopaminergic neurons are still present but the responses are now mediated solely by input from intrinsically photosensitive RGCs.

In mammals, five distinct dopamine receptors have been characterized, which are divided into two subfamilies based on their structure, pharmacology, and signaling properties. Dopamine D1-like receptors include D1 and D5 receptors and dopamine D2-like receptors include D2, D3, and D4 receptors [15]. Retinal D2 receptors are highly expressed in dopaminergic neurons [16, 17]. Stimulation of these receptors inhibits the release of dopamine [18–22]. Recently, Li et al. [23] reported the presence of D4 mRNA in dopaminergic amacrine cells in the mouse retina, suggesting that these receptors may also play a role in regulating dopamine release in the retina. Given the evidence that dopaminergic amacrine cells in degenerate retinas are dysfunctional and the importance of D2 receptors, in particular, in regulating dopaminergic activity in the retina, the goal of the present study was to investigate the effects of dopamine D2-like receptor antagonists on the light responses of RGCs in the P23H rat, a well-characterized model of retinitis pigmentosa.

## Methods

### Ethical approval

This study was carried out in strict accordance with the recommendations in the Guide for the Care and Use of Laboratory Animals of the National Institutes of Health. The protocol was approved by the Institutional Animal Care and Use Committee of the VA Boston Healthcare System (Protocol Number: 304-J-063013). Every precaution was taken to minimize animal stress and the number of animals used. All surgery was performed in euthanized animals.

### Animals and tissue preparation

P23H-line 1 homozygous rats and Sprague-Dawley rats of 21–40 weeks of age were used in this study. At these ages, the outer nuclear layer of the P23H rat retina is greatly reduced with only a single row of (presumably) cone photoreceptors remaining [3, 24]. Breeding pairs of P23H-line 1 homozygous rats were generously donated by Dr. Matthew LaVail (University of California San Francisco, CA). Sprague-Dawley rats were obtained from Harlan Laboratories (Indianapolis, IN). The room light was kept on a 12 hr light/dark cycle using standard fluorescent lighting. During the light cycle, the illumination at the level of the cages was 100–200 lux.

On the day of an experiment, a rat was euthanized with sodium pentobarbital (150 mg/kg, i.p.), and the eyes were removed and hemisected under normal room light. After removal of the vitreous humour from each eye, one eyecup was transferred to a holding vessel containing bicarbonate-buffered Ames medium (Sigma-Aldrich), which was continuously gassed at room temperature with 5% CO_2_/95% O_2_. The retina of the other eyecup was gently peeled from the retinal pigment epithelium/choroid and trimmed into a square of ~ 12 mm^2^. The retina was then placed photoreceptor side down in a small-volume (0.1 ml) chamber. The chamber was mounted on a fixed-stage upright microscope (Nikon Eclipse E600FN), and the retina superfused at 1.5 ml/min with bicarbonate-buffered Ames medium supplemented with 2 mg/ml D-(+) glucose and equilibrated with 5% CO_2_/95% O_2_. An in-line heating device (Warner Instruments) was used to maintain recording temperature at 35–36°C. The retina of the other eyecup was used later in the day. All experiments were performed between 10 am and 5 pm.

### Electrophysiological recordings

Action potentials (spikes) were recorded extracellularly from individual RGCs. With the aid of red light (>630 nm) that was delivered from below the chamber, the tip of a glass-insulated platinum/tungsten microelectrode (0.6–1.0 MΩ impedance; Thomas Recording GmbH, Germany) was visually advanced to the retinal surface with a motor-driven micromanipulator. Extracellular potentials from RGCs were amplified and bandpass filtered at 100 to 5000 Hz by a differential amplifier (Xcell-3; FHC, Bowdoin, ME). To ensure that recordings were made from single cells, the recorded waveform of the spike was continuously displayed in real time on a PC to check for uniformity of spike size and shape. Spikes from single RGCs were converted to standard transistor to transistor logic (TTL) pulses with a time-amplitude window discriminator (APM Neural Spike Discriminator, FHC). A laboratory data acquisition system (1401 Processor and Spike2 software; Cambridge Electronic Design Ltd., Cambridge, UK) was used to digitize the TTL pulses and raw spike train data.

### Light stimulation

Light from a mercury arc lamp illuminated an aperture that was focused on the retina from above, through the 4X objective of the microscope. The image produced on the retina was either a 250-μm or 1.5-mm diameter spot, which was centered on the recorded RGC. In the light path was a 545 nm interference filter (bandwidth, 30 nm). The intensity of the unattenuated light stimulus on the retina, measured with a spectroradiometer (ILT900-R, International Light Technologies), was 8.5 x 10^17^ photons/cm^2^/s (~ 2 x 10^6^ lux). In this paper, the light intensities are expressed in log units relative to the unattenuated light intensity. An electromechanical shutter (Uniblitz, Rochester, NY) was used to control the stimulus duration, which was set to 100 ms in constructing intensity-response curves. During recordings from RGCs, light flashes were presented with interstimulus intervals of 3–15 s (depending upon the duration and intensity of the light stimulus) to avoid any adapting effect of the previous flash. All experiments were performed in a dimly lighted room (10 lux).

### Drugs

(–)-Sulpiride, (–)-eticlopride, and L-745,870 were purchased from Tocris Bioscience (Minneapolis, MN). Eticlopride and L-745,870 were dissolved in physiological saline (0.9% NaCl). Sulpiride was dissolved in dimethylsulphoxide (DMSO) and diluted 50-fold in physiological saline. The drug solutions were applied to the bathing solution using calibrated syringe pumps (Razel Scientific Instruments). The final concentration of DMSO at the retina for the sulpiride solution was approximately 0.02%. Drugs were bath applied for ~10 min to ensure stable responses before effects were examined. Only one cell was studied in each retina to avoid possible residual drug effects.

### Data analysis

Intensity-response curves were generated from the responses of RGCs to 100 ms flashes of light. The light responses of RGCs were calculated by counting the number of spikes within a 100 ms window that encompassed the peak response and subtracting any spontaneous activity, measured between light stimuli. Cell responses were averaged from 5 stimulus presentations. Intensity-response curves of RGCs were fitted with a sigmoidal dose-response (variable slope), using SigmaPlot 10.0 (SPSS, Chicago, IL). Three parameters were measured from the curve fits: maximum peak response, dynamic range, and light sensitivity. The maximum peak response was simply the result from the fit of data points. The dynamic range was defined as the range of light intensity that elicited responses between 10 and 90% of the maximum peak response. Drug-induced change in light sensitivity was determined by comparing the light intensity that evoked a half-maximum response prior to drug application with the light intensity that evoked the same peak response in the presence of the drug. Numerical data in the text and error bars in figures are expressed as the mean ± standard deviation. Statistical significance was carried out using two-tailed paired Student’s t-tests (SigmaStat 3.5 software), with P < 0.05 considered significant.

## Results

I will first report on the effects of dopamine D2-like receptor antagonists on intensity-response functions of RGCs to short duration (100 ms) flashes of light. I will then describe the effects of the dopamine D2-like receptor antagonists on the responses of an atypical ON-center RGC to long duration (500–700 ms) flashes of light.

Intensity-response curves from the responses of RGCs were generated from both ON-center and OFF-center RGCs to stimulation with small (250-μm diameter) or large (1.5-mm diameter) spots of light. Since the effects of the receptor antagonists on intensity-response curves of ON-center and OFF-center cells appeared similar regardless of whether the cells were stimulated with a small spot or large spot of light, the data were pooled in the overall analysis to improve statistical power. For those RGCs that were stimulated with *both* small and large spots of light, values were averaged before statistical analysis.

### Effects of dopamine D2/D3 receptor antagonists on intensity-response functions of Sprague-Dawley rat RGCs

I examined the effects of the D2/D3 receptor antagonists sulpiride (10–15 μM) and eticlopride (1 μM), which displays a greater selectivity than sulpiride for D2 and D3 receptors over D4 receptors [25], on the intensity-response functions of 11 Sprague-Dawley rat RGCs. Seven cells were treated with eticlopride and 4 cells were treated with sulpiride. The cells were stimulated with either a small spot (5 ON-center cells) or a large spot (4 OFF-center and 2 ON-center cells) of light.

**Fig 1:**
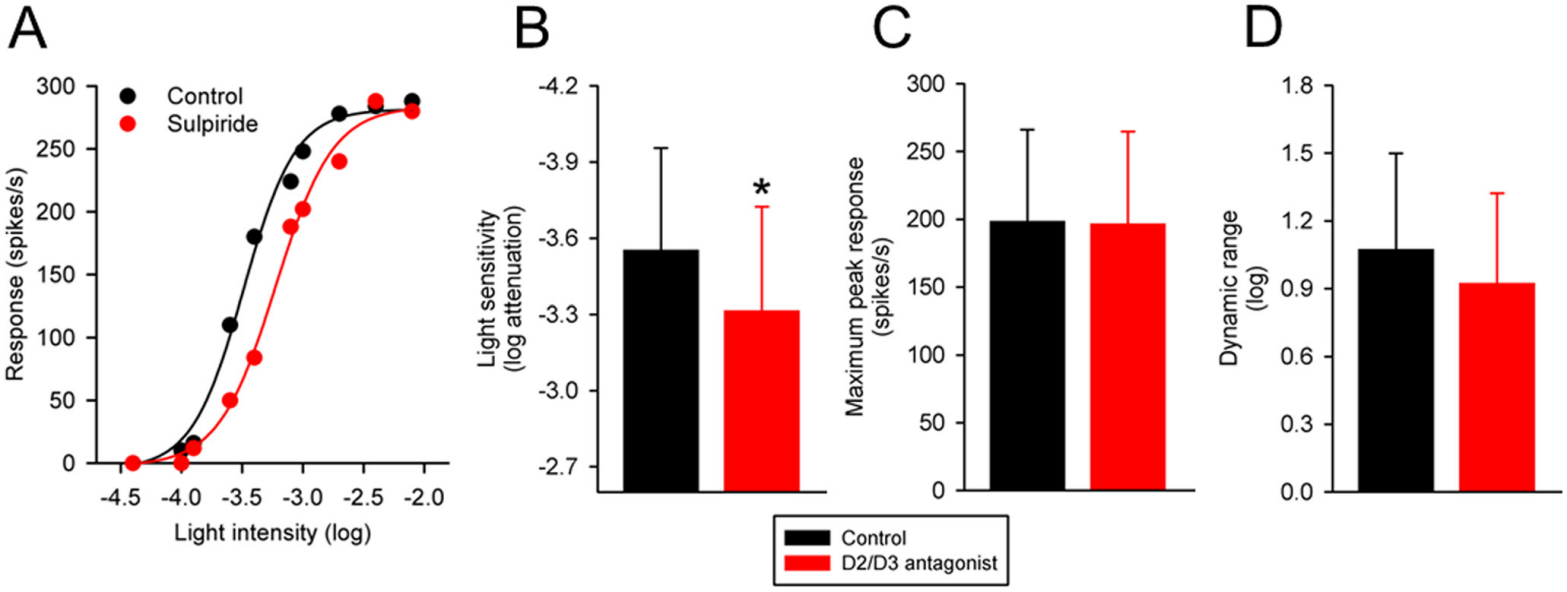
Effects of dopamine D2/D3 receptor antagonists on intensity-response curves of Sprague-Dawley rat RGCs. (A) Intensity-response curve from an ON-center Sprague-Dawley rat RGC to a small spot of light, taken before and during application of sulpiride (10 μM) to the bathing solution. The abscissa is labeled as log-unit attenuation in stimulus intensity from the maximum (8.5 x 10^17^ photons/cm^2^/s). Summary data illustrating the change induced by dopamine D2/D3 receptor antagonists in (B) light sensitivity, (C) maximum peak response, and (D) dynamic range of Sprague-Dawley rat RGCs (n = 11). Histogram data are expressed as the mean (SD). Asterisk indicates significantly different from control (P < 0.05).

Fig 1A shows the effects of a D2/D3 receptor antagonist on the intensity-response function of a representative Sprague-Dawley rat RGC. The light intensity that evoked a half-maximum response prior to application of sulpiride was –3.50 log units attenuation. With application of sulpiride, the light intensity that evoked the same response was –3.24 log units attenuation. By definition, sulpiride reduced the light sensitivity of the cell by 0.26 log unit. Sulpiride had little effect on either the maximum peak response or the dynamic range (the light intensity span that elicits responses between 10% and 90% of the maximum peak response) of this cell to the spot of light. Fig 1B, 1C and 1D summarize the effects of the D2/D3 receptor antagonists sulpiride and eticlopride on the light sensitivity, maximum peak response and dynamic range of the RGCs (n = 11). On average, the D2/D3 receptor antagonists decreased light sensitivity of the RGCs by 0.23 log unit (from 3.55 ± 0.40 log to 3.32 log ± 0.41 log units attenuation; P = 0.001). No statistically significant effect was observed in either the maximum peak response (P = 0.664) or the dynamic range (P = 0.117) of the RGCs. Taken together, the data suggest that blocking dopamine D2/D3 receptors in the healthy Sprague-Dawley rat retina simply shifts intensity-response curves of RGCs to the right.

### Effects of dopamine D2/D3 receptor antagonists on intensity-response functions of P23H rat RGCs

I examined the effects of sulpiride (10–15 μM) and eticlopride (1 μM) on the intensity-response functions of 16 P23H rat RGCs. Nine cells were treated with sulpiride and 7 cells were treated with eticlopride. The cells were stimulated with a small spot of light (1 ON-center and 2 OFF-center cells), a large spot of light (4 ON-center and 2 OFF-center cells), or both small and large spots of light (3 ON-center and 4 OFF-center cells).

**Fig 2:**
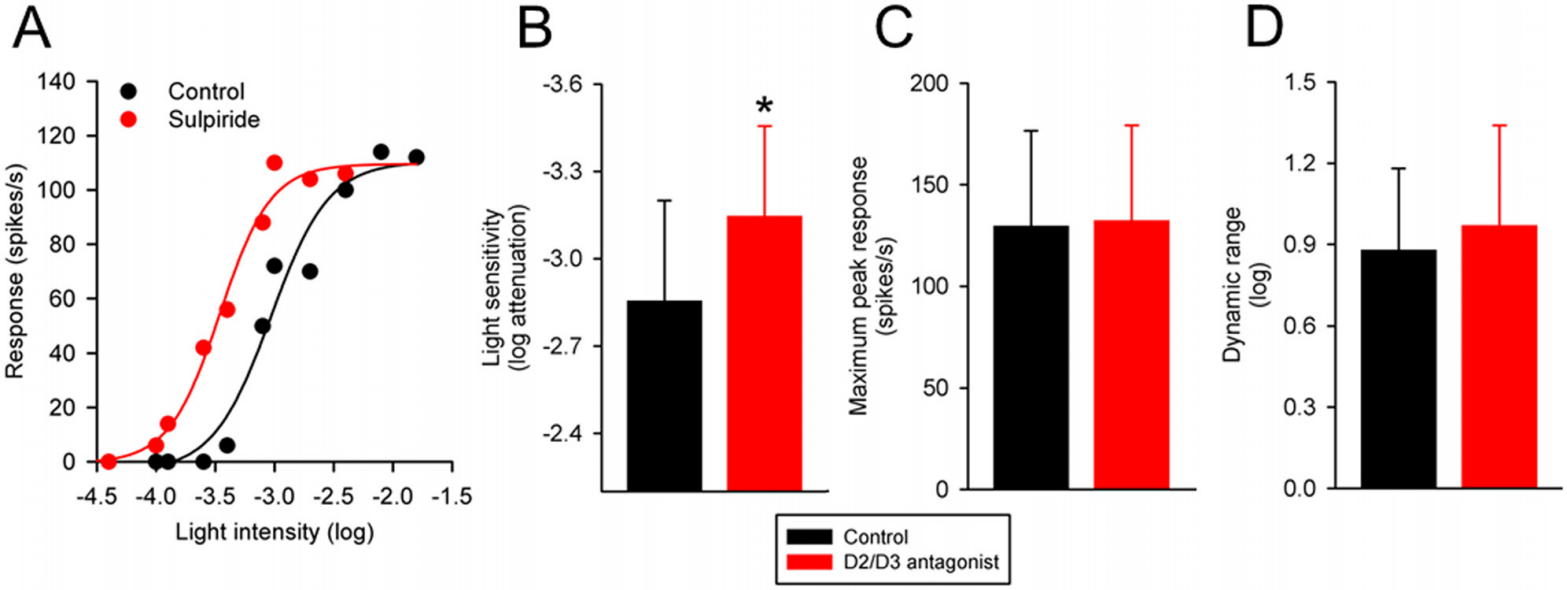
Effects of dopamine D2/D3 receptor antagonists on intensity-response curves of P23H rat RGCs. (A) Intensity-response curve from an ON-center P23H rat RGC to a large spot of light, taken before and during application of sulpiride (15 μM) to the bathing solution. The abscissa is labeled as log-unit attenuation in stimulus intensity from the maximum (8.5 x 10^17^ photons/cm^2^/s). Summary data illustrating the change induced by dopamine D2/D3 receptor antagonists in (B) light sensitivity, (C) maximum peak response, and (D) dynamic range of P23H rat RGCs (n = 16). Histogram data are expressed as the mean (SD). Asterisk indicates significantly different from control (P < 0.05).

Fig 2A shows the effects of a D2/D3 receptor antagonist on the intensity-response function of a representative P23H rat RGC. Sulpiride increased the light sensitivity of the RGC to the spot of light by 0.44 log unit. Sulpiride had little effect on either the maximum peak response or the dynamic range of this cell to the spot of light. Fig 2B, 2C and 2D summarize the effects of the D2/D3 receptor antagonists sulpiride and eticlopride on the light sensitivity, maximum peak response and dynamic range of the RGCs (n = 16). On average, the D2/D3 receptor antagonists increased light sensitivity of the RGCs by 0.29 log unit (from 2.86 ± 0.35 log to 3.15 log ± 0.31 log units attenuation; P<0.001). No statistically significant effect was observed in either the maximum peak response (P = 0.482) or the dynamic range (P = 0.206) of the RGCs. Taken together, the data suggest that blocking dopamine D2/D3 receptors in the P23H retina simply shifts intensity-response curves of RGCs to the left.

### Effects of a dopamine D4 receptor antagonist on intensity-response functions of Sprague-Dawley rat RGCs

Dopamine D3 receptors appear to be absent in the rat retina [17, 26], indicating that the observed effects of sulpiride and eticlopride were likely due to blockade of D2 receptors. The other D2-like receptor in the retina is the D4 receptor, which is highly expressed in photoreceptors [27, 28]. I examined the effects of L-745,870 (1 μM), a potent and selective dopamine D4 receptor antagonist [29], on the intensity-response functions of 14 Sprague-Dawley rat RGCs. The cells were stimulated with either a small spot (4 OFF-center and 2 ON-center cells) or a large spot (5 ON-center and 3 OFF-center cells) of light.

**Fig 3:**
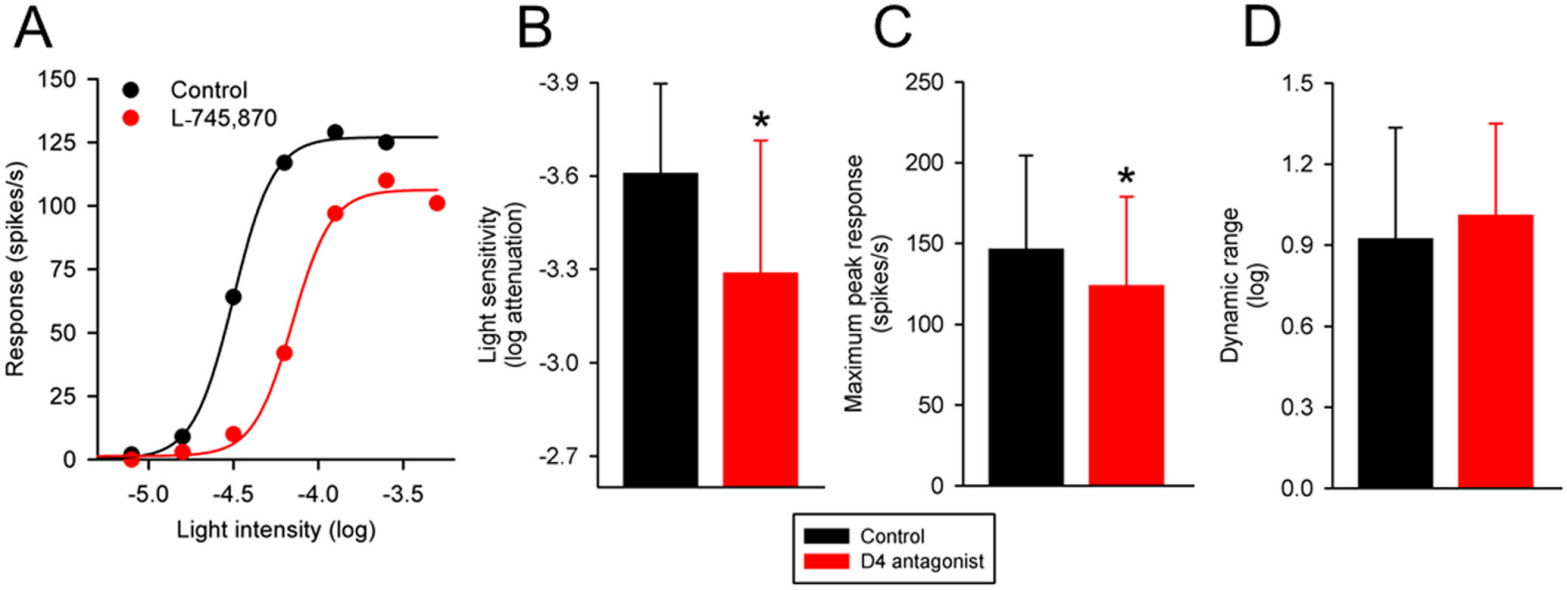
Effects of a dopamine D4 receptor antagonist on intensity-response curves of Sprague-Dawley rat RGCs. (A) Intensity-response curve from an ON-center Sprague-Dawley rat RGC to a large spot of light, taken before and during application of L-745,870 (1 μM) to the bathing solution. The abscissa is labeled as log-unit attenuation in stimulus intensity from the maximum (8.5 x 10^17^ photons/cm^2^/s). Summary data illustrating L-745,870-induced change in (B) light sensitivity, (C) maximum peak response, and (D) dynamic range of Sprague-Dawley rat RGCs (n = 14). Histogram data are expressed as the mean (SD). Asterisk indicates significantly different from control (P < 0.05).

Fig 3A shows the effects of the dopamine D4 receptor antagonist on the intensity-response function of a representative Sprague-Dawley rat RGC. The light intensity that evoked a half-maximum response prior to application of L-745,870 was –4.50 log units attenuation. With application of L-745,870, the light intensity that evoked the same response was– 4.11 log units attenuation. L-745,870 had decreased the light sensitivity of the cell by 0.39 log unit. The maximum peak response of the cell to stimulation the spot of light decreased from 127 to 106 spikes/s. L-745,870 had no effect on the dynamic range of this cell to the spot of light. Fig 3B, 3C and 3D summarize the effects of L-745,870 on the light sensitivity, maximum peak response and dynamic range of the RGCs (n = 14). On average, L-745,870 decreased light sensitivity of the RGCs by 0.32 log unit (from 3.61 ± 0.29 to 3.29 ± 0.43 log units attenuation; P<0.001) and decreased the maximum peak response of the RGCs from 147 ± 58 to 124 ± 55 spikes/s (P = 0.007). L-745,870 had no statistically significant effect on the dynamic range of the RGCs (P = 0.392). Taken together, the data suggest that blocking dopamine D4 receptors in the Sprague-Dawley rat retina shifts intensity-response curves of RGCs downward and to the right.

### Effects of a dopamine D4 receptor antagonist on intensity-response functions of P23H rat RGCs

I examined the effects of L-745,870 (1 μM) on the intensity-response functions of 12 P23H rat RGCs. The cells were stimulated with a small spot of light (3 ON-center cells), a large spot of light (1 ON-center and 2 OFF-center cells), or both small and large spots of light (3 ON-center and 3 OFF-center cells).

**Fig 4:**
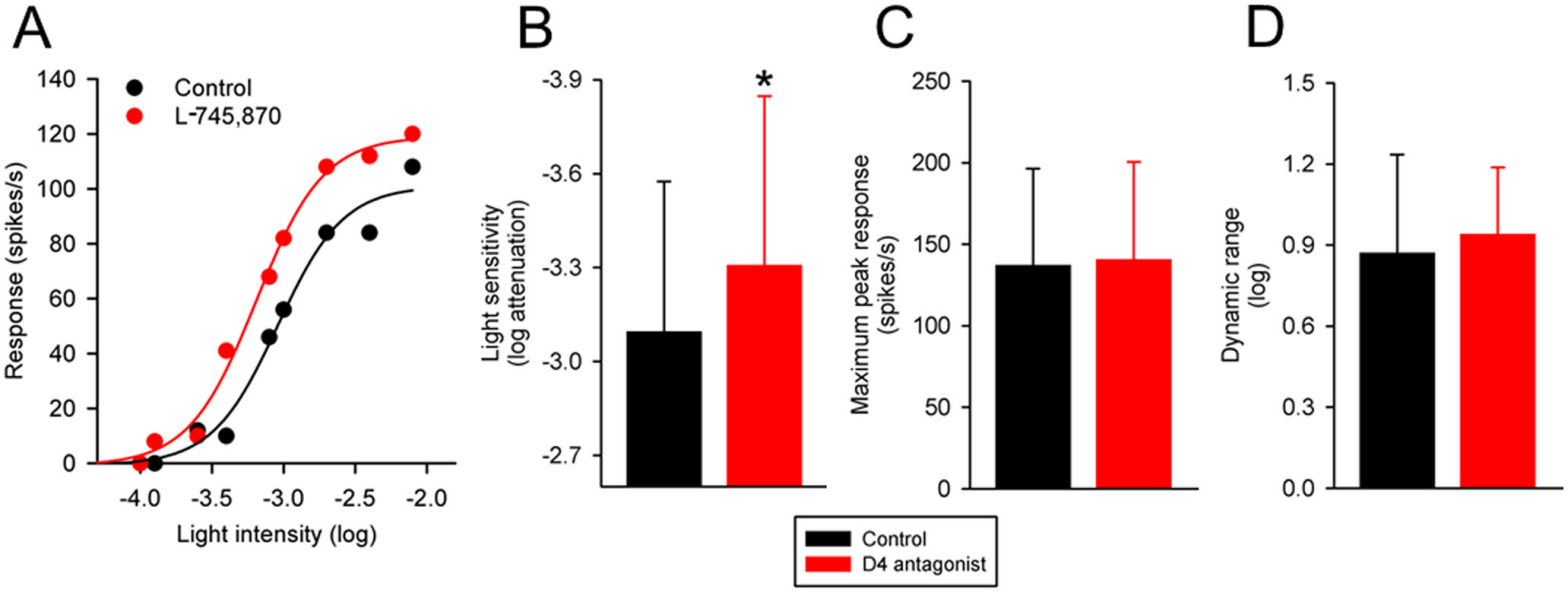
Effects of a dopamine D4 receptor antagonist on intensity-response curves of P23H rat RGCs. (A) Intensity-response curve from an OFF-center P23H rat RGC to a small spot of light, taken before and during application of L-745,870 (1 μM) to the bathing solution. The abscissa is labeled as log-unit attenuation in stimulus intensity from the maximum (8.5 x 10^17^ photons/cm^2^/s). Summary data illustrating L-745,870-induced change in (B) light sensitivity, (C) maximum peak response, and (D) dynamic range of P23H rat RGCs (n = 12). Histogram data are expressed as the mean (SD). Asterisk indicates significantly different from control (P < 0.05).

Fig 4A shows the effects of the dopamine D4 receptor antagonist on the intensity-response function of a representative P23H rat RGC. The light intensity that evoked a half-maximum response prior to application of L-745,870 was –3.04 log units attenuation. With application of L-745,870, the light intensity that evoked the same response was– 3.26 log units attenuation. L-745,870 had increased the light sensitivity of the cell by 0.22 log unit. Fig 4B, 4C and 4D summarize the effects of L-745,870 on the light sensitivity, maximum peak response and dynamic range of the RGCs (n = 12). On average, L-745,870 increased light sensitivity of the RGCs by 0.21 log unit (from 3.10 ± 0.48 log to 3.31 log ± 0.54 log units attenuation; P<0.001). No statistically significant effect was observed in either the maximum peak response (P = 0.274) or the dynamic range (P = 0.508) of the RGCs. Taken together, these data suggest that blocking dopamine D4 receptors simply shifts intensity-response curves of P23H rat RGCs to the left.

### Effects of dopamine D2-like receptor antagonists on the light responses of an atypical ON-center P23H rat RGC

**Fig 5:**
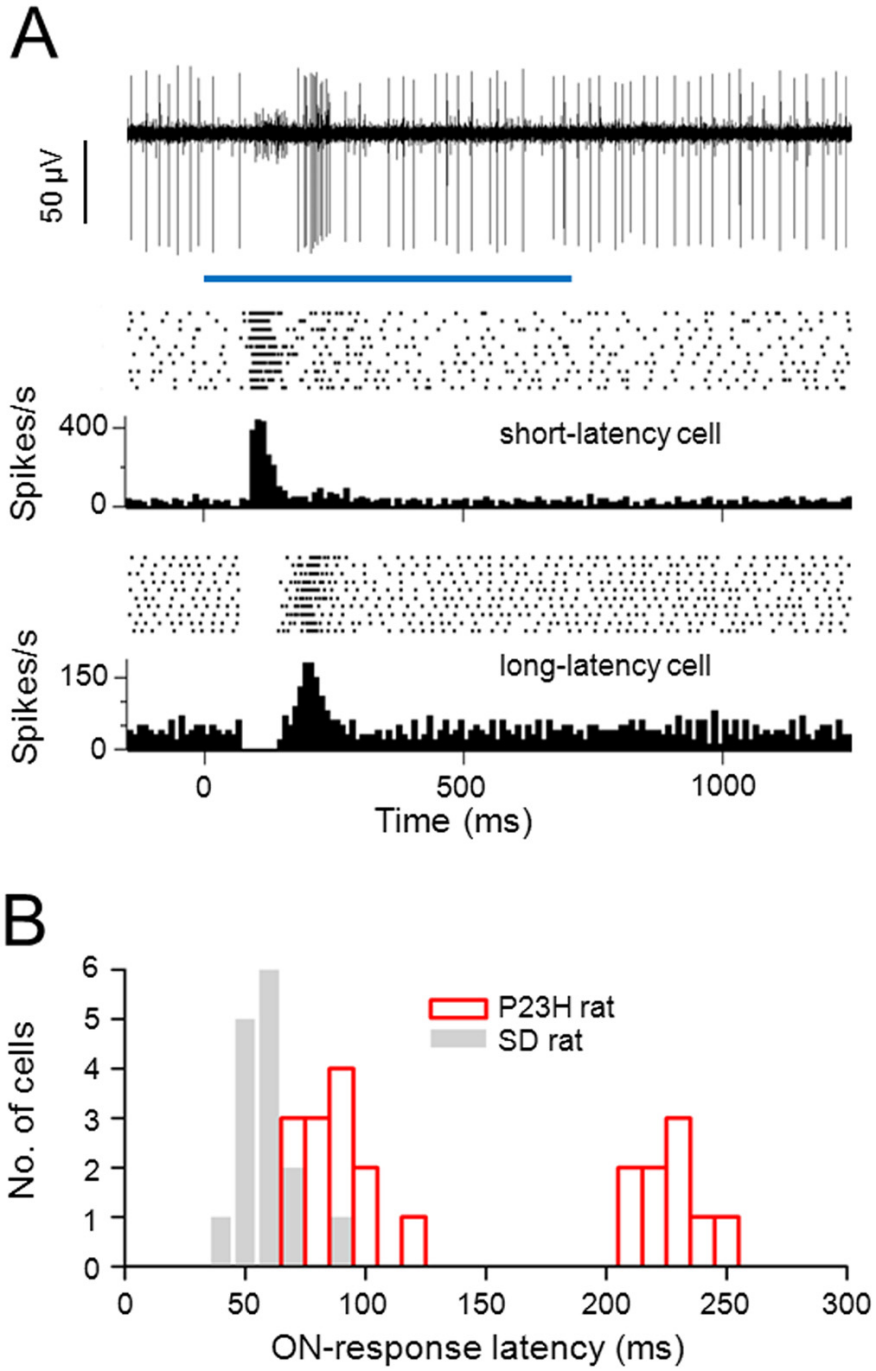
Light responses of two types of ON-center P23H rat RGC. (A) Top: extracellular recording of the responses from both a short-latency and a long-latency ON-center RGC to a 700 ms flash of a small spot of light (indicated by the blue horizontal bar). Bottom: spike rasters and PSTHs of spike activity recorded from the two RGCs. (B) Distribution of response latencies from ON-center P23H rat RGCs (n = 22) and ON-center Sprague-Dawley (SD) rat RGCs (n = 15). Response latencies of RGCs were measured from flashes of light (small spot) that evoked a maximum or near-maximum peak response. Response latency was defined as the time after light onset to the peak firing frequency (10 ms bin width) (cf. [30]).

All RGCs in this study were identified as either ON-center or OFF-center cells from their responses to a 500–700 ms flash of a small (250-μm diameter) spot of light centered over the receptive field. ON-center cells elicit an excitatory response to the onset but not to the offset of the spot of light, whereas OFF-center cells elicit an excitatory response to the offset of the spot of light. I found that two classes of ON-center P23H rat RGC could be distinguished based on the latency of the response to light onset. Fig 5A shows an extracellular recording where both types of ON-center cells were recorded simultaneously to stimulation with a small spot of light. The small-amplitude spiking cell responded with a 65 ms burst of spikes, beginning 90 ms after light onset, while the large-amplitude spiking cell responded with a 60 ms burst of spikes, beginning 170 ms after light onset. Note the cessation of spike activity of the large-amplitude spiking cell immediately following the onset of light, during which time the short-latency ON-center cell had the transient peak in activity. This reduction in spike activity can also be appreciated in the bottom raster plot and peristimulus time histogram (PSTH). A reduction in spike activity at light onset was a consistent finding among long-latency ON-center cells.

Fig 5B shows the distribution of response latencies of the 22 ON-center P23H rat RGCs recorded in this study. Of these, 9 cells showed a long-latency response. The distribution of response latencies of P23H rat RGCs was clearly bimodal. On average, the latency of the short-latency ON responses was 86.9 ± 14.4 ms, and the latency of the long-latency ON responses was 227 ± 13.2 ms. Response latencies of 15 ON-center Sprague-Dawley rat RGCs recorded are also shown in this figure for comparison. The response latency of ON-center Sprague-Dawley rat RGCs was on average 58.7 ± 11.9 ms. Long-latency ON-center cells were not observed in Sprague-Dawley rat retinas.

**Fig 6:**
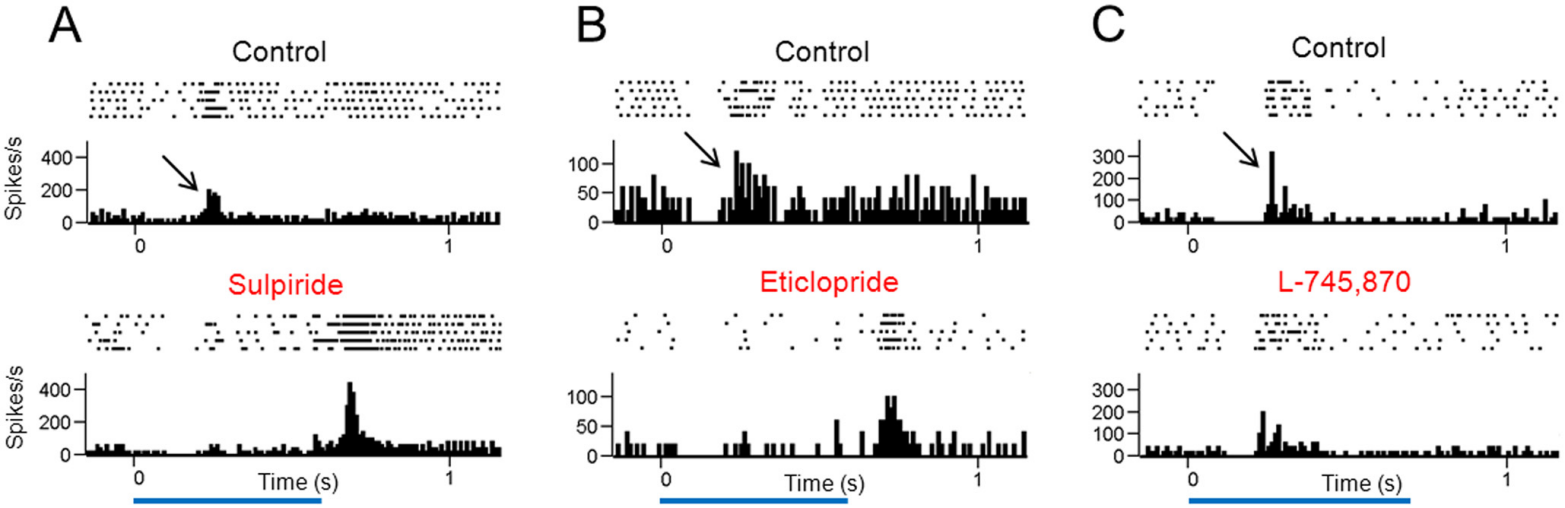
Effects of dopamine D2-like receptor antagonists on light responses of long-latency ON-center P23H rat RGCs. Displayed are spike rasters and PSTHs of spike activity recorded from three RGCs to stimulation with a small spot of light centered over the receptive field. The D2/D3 receptor antagonists sulpiride (panel A) and eticlopride (panel B) eliminated the ON responses (indicated by arrows) and brought out OFF responses. The D4 receptor antagonist L-745,870 (panel C) had very little effect on the light response. Blue horizontal bars indicate the duration of the light stimulus.

Of the 9 long-latency ON-center RGCs, 3 cells were tested with sulpiride, 3 cells were tested with eticlopride, and 3 cells were tested with L-745,870. For the cells that were examined with the D2/D3 receptor antagonists sulpiride and eticlopride, the long-latency ON responses (calculated with a 200-ms window encompassing the peak activity and subtracting the pre-stimulus spontaneous activity) to a small spot of light were significantly reduced (from 30.5 ± 12.2 to 4.5 ± 7.5 spikes/s, P = 0.009, n = 6). Along with this reduction in the ON response, a short-latency (95.0 ± 12.3 ms) OFF response appeared in all 6 cells. This OFF response (calculated with a 200-ms window encompassing the peak activity and subtracting the pre-stimulus spontaneous activity) varied from 25 to 94 spikes/s (mean, 48.5 spikes/s). Fig 6A and 6B show the light responses of one long-latency ON-center RGC that was treated with sulpiride and another long-latency ON-center RGC that was treated with eticlopride. In both cases, the ON response disappeared while an OFF response appeared. Neither sulpiride nor eticlopride brought out an OFF response in short-latency ON-center P23H rat RGCs (data not shown). For the 3 cells that were tested with the D4 receptor antagonist L-745,870, the ON response persisted (29.3 ± 2.5 spikes/s in control versus 29.0 ± 9.5 spikes/s in the presence of L-745,870, P = 0.910). This is illustrated for one long-latency ON-center RGC in Fig 6C.

**Fig 7:**
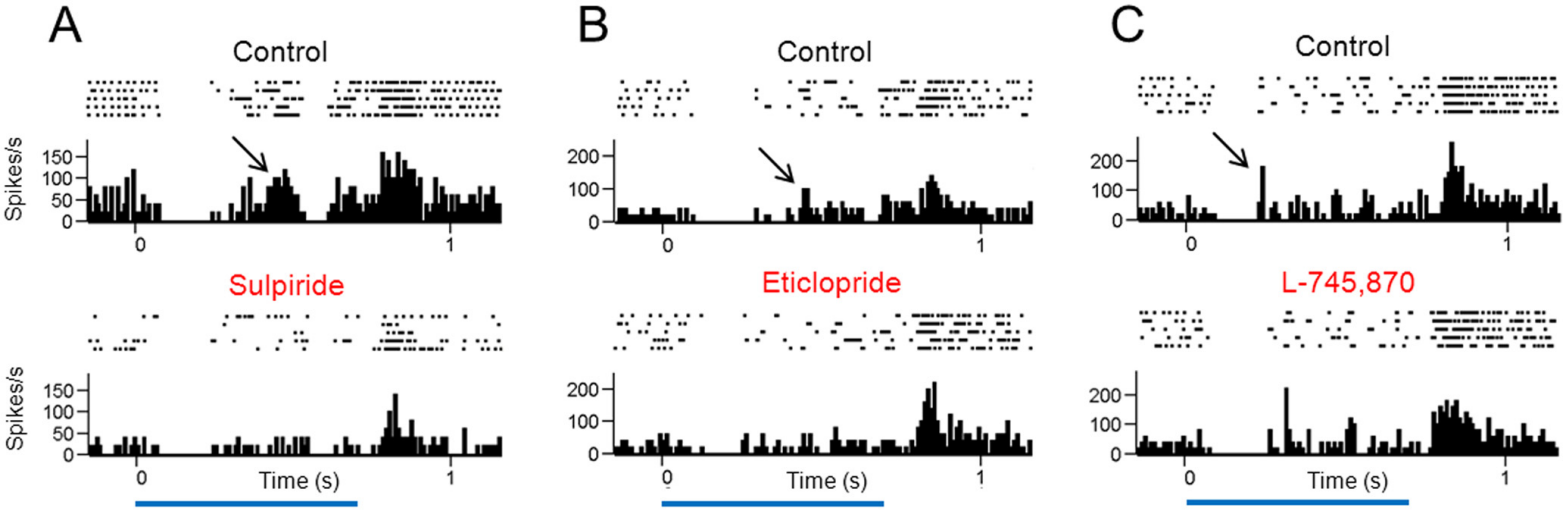
Effects of dopamine D2-like receptor antagonists on light responses of OFF-center P23H rat RGCs that showed a long-latency ON response. Displayed are spike rasters and PSTHs of spike activity recorded from three RGCs to stimulation with a small (250-μm diameter) spot of light centered over the receptive field. Sulpiride and eticlopride, but not L 745,870, diminished the delayed ON responses (indicated by arrows). Blue horizontal bars indicate the duration of the light stimulus.

An intriguing possibility is that long-latency ON-center RGCs were at one time OFF-center cells early in the disease process. In support of this idea I observed that some OFF-center P23H rat RGCs showed a long-latency ON response when stimulated with a small spot of light. Furthermore, when the cells were exposed to either sulpiride (n = 2 cells) or eticlopride (n = 2 cell), but not L-745,870 (n = 3 cells), the ON responses were selectively reduced (Fig 7). The D2/D3 receptor antagonists sulpiride and eticlopride reduced the long-latency ON responses from 16.5 ± 4.8 to 2.3 ± 1.5 spikes/s (P = 0.004, n = 4). In the presence of the D4 receptor antagonist L-745,870, the long-latency ON responses remained intact (20.3 ± 3.1 spikes/s in control versus 21.7 ± 4.2 spikes/s in the presence of L-745,870, P = 0.742, n = 3). Whether long-latency ON-center RGCs were at one time OFF-center cells early in the disease process will need to be confirmed in future studies.

### Effects of dopamine D2-like receptor antagonists on spontaneous activity of Sprague-Dawley and P23H rat RGCs

How do the dopamine D2-like receptor antagonists used in this study affect spontaneous activity of the Sprague-Dawley and P23H rat RGCs? Table 1 shows the effects of the dopamine D2/D3 antagonists sulpiride and eticlopride and the dopamine D4 antagonist L-745,870 on the spontaneous activity of OFF-center and ON-center Sprague-Dawley rat RGCs. In general, the spontaneous activity in OFF-center cells was higher than in ON-center cells. There were no significant effects of the dopamine D2/D3 receptor antagonists on the spontaneous activity of either OFF-center cells or ON-center cells. The dopamine D4 antagonist L-745,870 reduced the spontaneous activity of OFF-center cells by 28% but had no significant effect on the spontaneous activity of ON-center cells.

**Table 1:** Effects of Dopamine D2-like Receptor Antagonists on Spontaneous Activity of Sprague-Dawley Rat RGCs.

	Control (spikes/s)	Drug (spikes/s)	No. of cells	P-value
**Sulpiride/Eticlopride**				
OFF cells	41.3±16.9	48.1±6.3	4	0.463
ON cells	12.2±8.3	19.7±22.5	7	0.426
**L-745,870**				
OFF cells	22.7±7.6	16.3±8.4	7	0.018
ON cells	10.7±17.7	10.9±17.1	7	0.727

Table 2 shows the effects of the dopamine D2/D3 antagonists sulpiride and eticlopride and the dopamine D4 antagonist L-745,870 on the spontaneous activity of OFF-center and ON-center P23H rat RGCs. OFF-center and long-latency ON-center RGCs showed a much higher level of spontaneous activity than short-latency ON-center RGCs. It is noteworthy that Sekirnjak et al. [31] found that the spontaneous firing rate of ON-center and OFF-center P23H rat RGCs were on average 1.8 spikes/s and ~ 25 spikes/s, respectively. Their findings agree well with my data, if one assumes that the ON-center RGCs they had analyzed in their study were short-latency ON-center cells. Statistically, the observed changes in spontaneous activity that occurred in the presence of the dopamine receptor antagonists were not significant.

**Table 2:** Effects of Dopamine D2-like Receptor Antagonists on Spontaneous Activity of P23H Rat RGCs.

	Control (spikes/s)	Drug (spikes/s)	No. of cells	P-value
**Sulpiride/Eticlopride**				
OFF cells	30.9±9.7	25.5±9.2	6	0.112
Short-latency ON cells	0.4±0.5	1.9±1.6	5	0.135
Long-latency ON cells	33.9±13.6	20.5±7.2	6	0.066
**L-745,870**				
OFF cells	20.7±6.1	23.5±6.9	6	0.058
Short-latency ON cells	2.0±1.7	5.9±7.9	5	0.267
Long-latency ON cells	29.7±27.1	20.8±13.8	3	0.400

## Discussion

The principal findings of this study are that the dopamine D2/D3 receptor antagonists sulpiride and eticlopride and the dopamine D4 receptor antagonist L-745,870 increase light sensitivity of P23H rat RGCs but decrease light sensitivity of healthy Sprague-Dawley rat RGCs. I also report for the first time an atypical ON-center RGC in the P23H rat retina that produces a long-latency ON response to the flash of a small spot of light centered over the receptive field. Both sulpiride and eticlopride, but not L-745,870, diminish the ON response in these cells and unmask a short-latency OFF response. Since dopamine D3 receptors do not appear to be present in the retina [17, 26], the effects of sulpiride and eticlopride on RGC responses are likely the result of blocking of dopamine D2 receptors.

Previously, I reported that the GABA_C_ receptor antagonist TPMPA, like the dopamine D2-like receptor antagonists, increases the light sensitivity of RGCs in P23H rat retina and decreases the light sensitivity of RGCs in Sprague-Dawley rat retina [32]. The similarity in effects suggests a common pathway by which the dopaminergic and GABAergic systems converge onto the RGCs. GABA_C_ receptors are located primarily on rod and cone bipolar cells [33–35]. Although dopamine D2 receptors have not been found to my knowledge on bipolar cells, many bipolar cells express dopamine D1 receptors [36, 37]. In the tiger salamander retina, Wellis and Werblin [38] reported that in the presence of GABA both dopamine and a D1 receptor agonist reduce GABA_C_ receptor sensitivity at bipolar cell terminals. It is plausible that blocking D2 autoreceptors on dopaminergic amacrine cells in P23H rat retina increases extracellular dopamine levels sufficiently to activate dopamine D1 receptors on bipolar cells to reduce the GABA_C_ receptor-mediated inhibition. A recent study in the mouse retina found evidence that dopamine D4 receptors may be expressed in dopaminergic amacrine cells [23]. If these receptors function like D2 receptors to regulate dopamine release, then this could explain the similarity in the effects of the dopamine D2 and D4 receptor antagonists on light sensitivity of RGCs.

There is still the unanswered question of why the effect of the dopamine receptor antagonists on light sensitivity of RGCs in Sprague-Dawley rats is opposite to that found in P23H rats. To answer this question we need to understand the cellular and sub-cellular changes that occur in neurons in degenerate retinas. With the loss of photoreceptors, substantial remodeling occurs early in the disease process in other retinal neurons [2–10], including alterations in the expression of neurotransmitter receptors. In degenerate mouse and rat retinas, metabotropic glutamate receptor mGluR6s in rod bipolar cells have been found to be displaced from the dendrites to the cells bodies [2–5, 7]. Alterations in the expression of glutamate and GABA receptors in the inner retina of degenerate retinas have also been described. Gründer et al. [39] reported differences in the expression pattern of distinct NMDA receptor subunits between wild-type and dystrophic RCS retinas, and Srivastava et al. [10] reported higher levels of AMPA, glycine and GABA_A_ receptors and lower levels of GABA_C_ receptors in the rd1 mouse compared to wild-type mouse. Interestingly, Nguyen-Legros et. al [37] have reported that the expression of D1 receptors in the inner sublayer of the inner plexiform layer, where the axon terminals of rod bipolar cells are located, is noticeably absent in the dystrophic RCS rat retina. Given these modifications in receptor expression, it is not surprising that the effects of pharmacological agents on light responses of RGCs in degenerate retinas would differ from those in wild-type retinas. Further studies are however needed to explain the paradoxical effect of the neurotransmitter receptor antagonists on light sensitivity of RGCs in P23H and Sprague-Dawley rats.

In this study, I found an unusual type of RGC in P23H rat retina–a RGC that exhibits a long-latency ON response in response to a flash of a small spot of light over the receptive field center. These cells can easily be missed if the retina is stimulated with a brief (e.g., 100 ms) flash of light, in which case these long-latency cells could appear to be OFF-center cells. Although in my previous electrophysiological studies in the P23H rat retina [32, 40] I used long-duration flashes at the beginning of an experiment to classify cells as either ON- or OFF-center, it was assumed at the time that these long-latency cells were ‘unhealthy’ ON-center cells, recorded in areas of the retina that had perhaps undergone a greater degree of degeneration. The finding in the present study that the D2/D3 receptor antagonists sulpiride and eticlopride transform long-latency ON-center cells into OFF-center cells suggests that these cells might have at one time been actually OFF-center cells. In retrospect this would account for the observations in my earlier studies [32, 40] that OFF-center cells were encountered less frequently than ON-center cells in P23H rat retinas.

OFF-center RGCs in wild-type animals can generate long-latency ON responses under specific pharmacological conditions. Renteria et al. [30] reported that blockage of ON bipolar cell signaling in mouse retinas with the mGluR6 agonist APB (known also as AP4) unmasks a long-latency ON response in OFF-center RGCs. Their data indicate that the long-latency ON responses are driven by OFF pathway inputs to the RGCs that are normally suppressed by the ON pathway. Farajian et al. [41] reported that blocking *both* GABA_A_ and GABA_C_ receptors in the rabbit and mouse retinas unmasks an ON response in OFF-center RGCs. Interestingly, the ON responses in their study were *abolished* by AP4, suggesting a different retinal pathway is involved. It will be of interest to examine the effects of AP4 on the long-latency ON responses in P23H rat RGCs to get a better understanding of the source of these delayed ON responses.

Targeting the dopaminergic system in the retina may be useful in treating some retinal degenerative disorders. In a recent study conducted by Aung et al. [42], diabetic mice show improved visual functions when treated with L-DOPA, a dopamine precursor, or dopamine D1 and D4 receptor agonists. My data suggest that inhibition of dopamine D2-like receptors may be a clinically important mechanism for symptomatic treatment of retinitis pigmentosa. Current approaches in treatment of retinitis pigmentosa include electrical stimulation with microelectrode arrays, optogenetics, gene therapy, and stem cell treatments. Dopamine D2-like receptor antagonists may be especially interesting in combination with these other therapies.
